# Correlation of clinical and physical-technical image quality in chest CT: a human cadaver study applied on iterative reconstruction

**DOI:** 10.1186/s12880-015-0075-y

**Published:** 2015-08-19

**Authors:** An De Crop, Peter Smeets, Tom Van Hoof, Merel Vergauwen, Tom Dewaele, Mathias Van Borsel, Eric Achten, Koenraad Verstraete, Katharina D’Herde, Hubert Thierens, Klaus Bacher

**Affiliations:** Department of Basic Medical Sciences, Ghent University, Proeftuinstraat 86, B-9000 Ghent, Belgium; Department of Radiology, Ghent University Hospital, De Pintelaan 185, B-9000 Ghent, Belgium

**Keywords:** Chest CT, Image quality, Iterative reconstruction, Human cadaver study, Visual grading analysis

## Abstract

**Background:**

The first aim of this study was to evaluate the correlation between clinical and physical-technical image quality applied to different strengths of iterative reconstruction in chest CT images using Thiel cadaver acquisitions and Catphan images. The second aim was to determine the potential dose reduction of iterative reconstruction compared to conventional filtered back projection based on different clinical and physical-technical image quality parameters.

**Methods:**

Clinical image quality was assessed using three Thiel embalmed human cadavers. A Catphan phantom was used to assess physical-technical image quality parameters such as noise, contrast-detail and contrast-to-noise ratio (CNR).

Both Catphan and chest Thiel CT images were acquired on a multislice CT scanner at 120 kVp and 0.9 pitch. Six different refmAs settings were applied (12, 30, 60, 90, 120 and 150refmAs) and each scan was reconstructed using filtered back projection (FBP) and iterative reconstruction (SAFIRE) algorithms (1,3 and 5 strengths) using a sharp kernel, resulting in 24 image series. Four radiologists assessed the clinical image quality, using a visual grading analysis (VGA) technique based on the European Quality Criteria for Chest CT.

**Results:**

Correlation coefficients between clinical and physical-technical image quality varied from 0.88 to 0.92, depending on the selected physical-technical parameter. Depending on the strength of SAFIRE, the potential dose reduction based on noise, CNR and the inverse image quality figure (IQF_inv)_ varied from 14.0 to 67.8 %, 16.0 to 71.5 % and 22.7 to 50.6 % respectively. Potential dose reduction based on clinical image quality varied from 27 to 37.4 %, depending on the strength of SAFIRE.

**Conclusion:**

Our results demonstrate that noise assessments in a uniform phantom overestimate the potential dose reduction for the SAFIRE IR algorithm. Since the IQF_inv_ based dose reduction is quite consistent with the clinical based dose reduction, an optimised contrast-detail phantom could improve the use of contrast-detail analysis for image quality assessment in chest CT imaging. In conclusion, one should be cautious to evaluate the performance of CT equipment taking into account only physical-technical parameters as noise and CNR, as this might give an incomplete representation of the actual clinical image quality performance.

## Background

The number of CT examinations has increased rapidly over the last few years, resulting in a substantial increase in radiation dose of the population in the Western world [1]. It has been estimated that these CT examinations may be responsible for approximately 2 % of all incident cancer cases in the United States [2]. Consequently, a lot of efforts have been made over the last decade to reduce the radiation dose for the patient by introducing new techniques such as automatic tube current modulation, adaptive collimation and iterative reconstruction [3–6]. If new dose reduction techniques are implemented, the impact on the image quality has to be investigated.

Medical physicists assess the image quality in CT using technical phantoms, evaluating parameters as noise, modulation transfer function (MTF), contrast-to-noise ratio (CNR) and/or contrast-detail. However, as these phantom models are not related to patient anatomy, it is unclear whether this methodology is appropriate to evaluate the clinical image quality. Particularly for noise, this can be problematic, since noise measurements in a uniform phantom don’t account for the complex relationship between anatomical variability and image quality [7]. To be able to compare the performance of different CT scanners or to evaluate dose optimisation tools, it is of critical importance that physical-technical image quality based dose optimisation performance is related to the clinical image quality based dose optimisation performance.

Clinical image quality is typically assessed by applying a visual grading analysis (VGA) [8] or a receiver operating characteristics (ROC) [9] study setup in a patient population. However, these patient studies are rather difficult to implement since either large numbers of patient images must be available or one patient has to be exposed to different dose settings, which should be avoided from ethical point of view. As an alternative, clinical images of an anthropomorphic phantom can be acquired. Compared to physical-technical phantoms, these phantoms approximate better the clinical reality with respect to anatomical features [10].

In present study, patient image quality of chest CT was assessed by means of human cadavers, conserved using the Thiel embalming technique [11]. In contrast to the classical formol embalming technique, the Thiel embalming method results in excellent preservation of the flexibility and plasticity of organs and tissues [11, 12]. As a result, lungs can be inflated during image acquisition to simulate the anatomy of a chest CT [13]. Consequently, these Thiel embalmed cadavers are an excellent model to investigate the link between clinical and physical-technical image quality. This link was already established in conventional chest radiography [13]. However, with respect to CT imaging, the correlation between clinical and physical-technical image quality was not yet examined.

The first aim of this study was to evaluate the correlation between clinical and physical-technical image quality applied to different strengths of iterative reconstruction in chest CT images using Thiel cadaver acquisitions and Catphan images. The second aim was to determine the potential dose reduction of iterative reconstruction compared to conventional filtered back projection based on different clinical and physical-technical image quality parameters.

## Methods

### Thiel embalmed cadavers

The use of human cadavers is in compliance with the Helsinki Declaration and fulfilled the requirements of the ethical committee of our institution (Ghent University, B67020095736). The cadavers were obtained from body donations to the department of Anatomy of Ghent University.

Three human cadavers (2 male, 1 female) were embalmed using the methodology of Prof. Em. Walther Thiel, Anatomisches Institut Karl-Franzens-Universität, Graz, Austria [12].

**Fig. 1:**
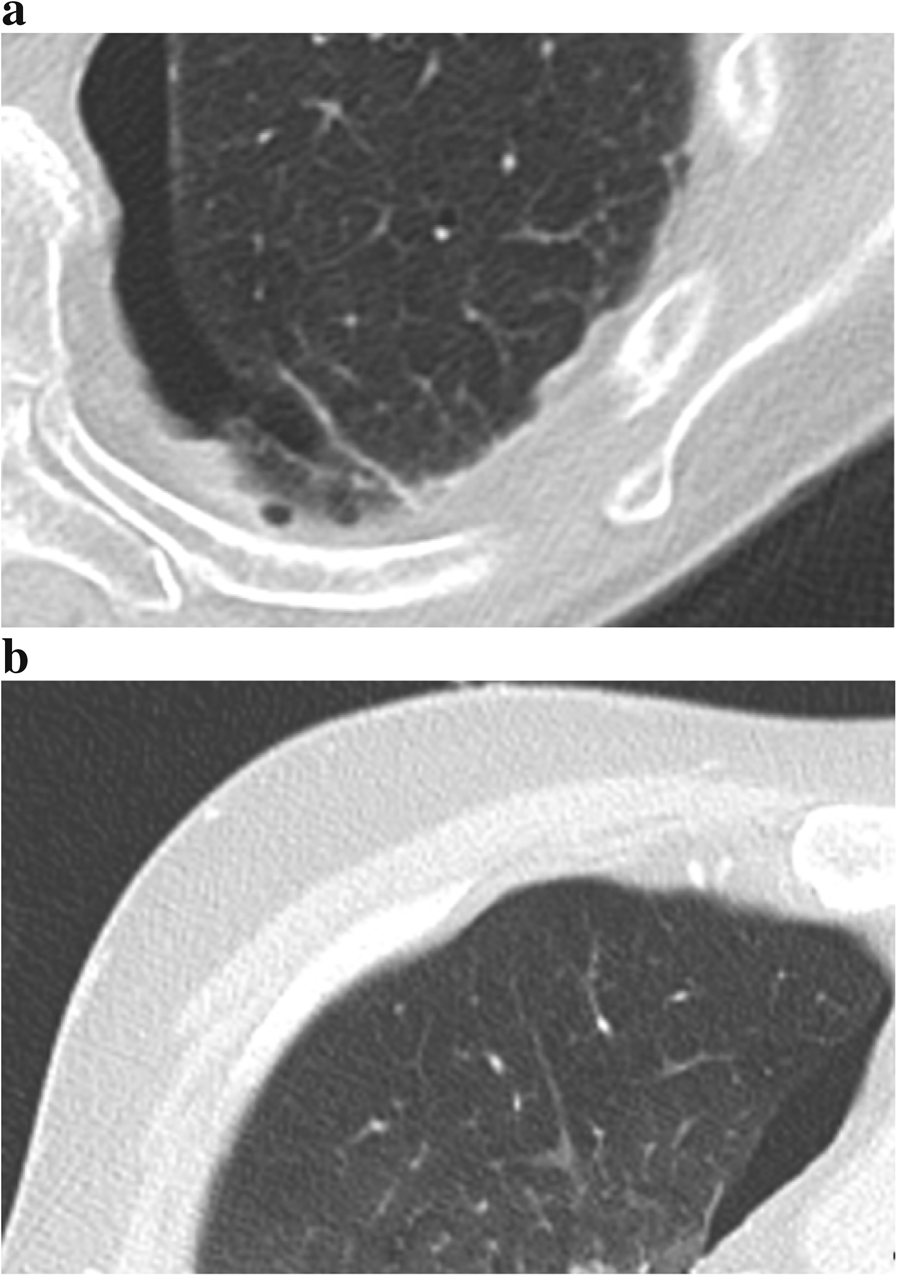
Patient versus Thiel cadaver chest CT image. Normal lung parenchyma illustrating nodular hypodense structures in a low density area, nodular hyperdense structures in a low density area, inter- or intralobular septa and the visceral pleura in both a patient (**a**) and a Thiel cadaver (**b**) chest CT image

Hereby, 4-chloro-3-methylenphenol as well as various salts are used for fixation and boric acid is added for disinfection. Furthermore, ethylene glycol is used for preservation of tissue plasticity, while the concentration of formalin is kept to the strict minimum (0.8 %) [11]. In contrast to standard formalin-embalmed human cadavers, this technique results in well preserved organs and tissues concerning colour, consistency, natural flexibility and natural plasticity. As a result, lung tissue is preserved completely which makes it possible to ventilate the lungs by performing a tracheotomy in combination with balloon ventilation. After ventilating the lungs, chest CT acquisitions can be acquired for subjective image quality analysis. In the cadavers used in this study, the lungs showed signs of pulmonary oedema and pulmonary parenchymal consolidation. Equivalency of patient and Thiel thoracic CT images is displayed in Fig. 1.

### Catphan phantom

**Fig. 2:**
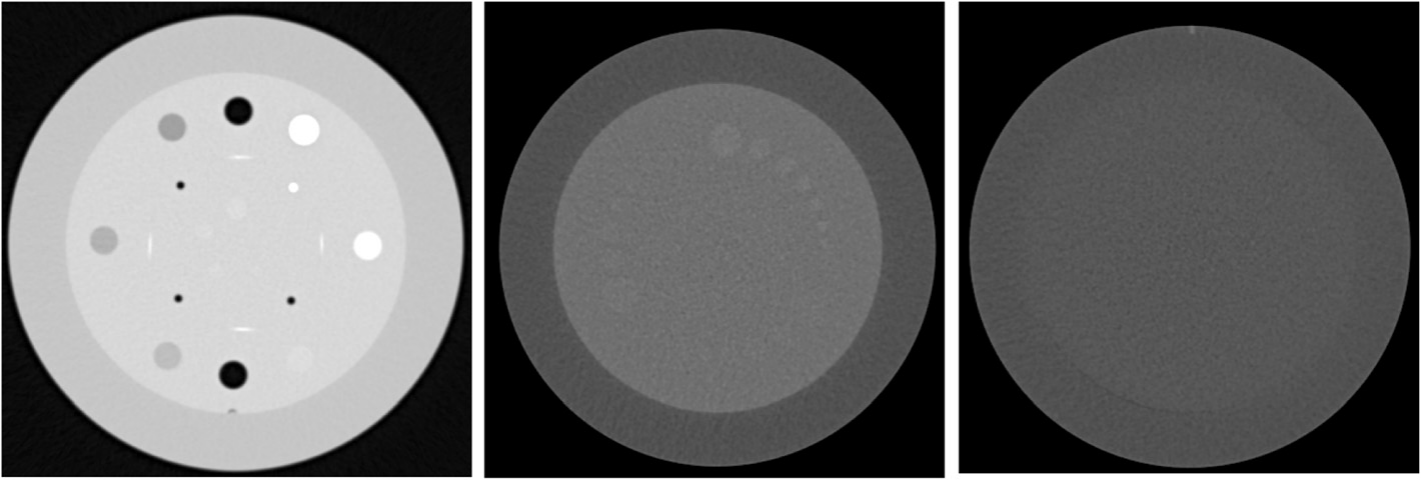
Catphan@504 phantom. The figure represents a CT image of the Catphan phantom. On the left, the CT number linearity and CT number accuracy module, which includes samples of teflon and acrylic used to calculate the CNR. In the middle, the low contrast module containing targets with different contrast levels: 1, 0.5 and 0.3 %. Each contrast level has 9 targets with different diameters: 15, 9, 8, 7, 6, 5, 4, 3, 2 mm. On the right the image uniformity module

To evaluate the physical-technical image quality the Catphan@504 phantom (The Phantom laboratory, Salem, New York, USA) was used. The phantom consists of several modules to evaluate high and low contrast resolution, CNR and noise (Fig. 2). In the low contrast module there are three areas with different contrast levels: 1, 0.5 and 0.3 %. Each contrast level contains targets with decreasing diameters (15, 9, 8, 7, 6, 5, 4, 3 and 2 mm). The CT number linearity and CT number accuracy module contains targets made from teflon, delrin, acrylic, polystyrene, low density polyethylene (LDPE), polymethylpentene (PMP) and air. The image uniformity module is made from a uniform material. The material’s CT number is designed to be within 2 % (20 HU) of water’s density at standard scanning protocols.

### Image acquisition

All images in this study were acquired with a Somatom Definition Flash CT scanner (Siemens Healthcare, Erlangen, Germany). The CT scanner is equipped with the dual source technology, CARE Dose4D, CARE kV, and Sinogram Affirmed Iterative Reconstruction (SAFIRE).

Chest CT scans of the lung ventilated Thiel embalmed cadavers were acquired using CARE Dose4D at different reference mAs values (12, 30, 60, 90, 120 and 150 refmAs), resulting in a mean CTDI_vol_ of 0.84, 2.05, 4.08, 6.18, 8.35 and 11.59 mGy respectively. The 90 refmAs setting is clinically applied in our institution. Other scan parameters were 120 kVp and pitch 0.9. Each data set was reconstructed at 3 mm using filtered back projection (FBP) with a sharp kernel (B70). To compare the FBP and the SAFIRE technique, all six data sets were also reconstructed using different strengths of IR (1,3 and 5 iteration steps). Similarly to the FBP reconstructed images, IR images were reconstructed using a sharp kernel (I70-1, I70-3, I70-5), resulting in a total of 24 image series (6 refmAs settings with each 4 reconstruction settings).

Afterwards Catphan images were acquired without CARE Dose4D at a mAs value corresponding to the mean mAs value over the different slices in the Thiel cadaver acquired at the six different refmAs settings, resulting in a CTDIvol of 0.84, 2.11, 4.19, 6.37, 8.82 and 12.23 mGy respectively. The same reconstruction settings as for the Thiel embalmed cadavers were used. All scanning and reconstruction parameters and the investigated phantoms and image quality parameters are listed in Table 1.

**Table 1:** Scanning and reconstruction parameters, investigated phantoms and image quality parameters used in this study

Fixed scan parameters	CTDI_(vol)_	Reconstruction parameters for each CTDI_(vol)_	Scanned objects	Investigated image quality parameters	
	Thiel/Catphan				
				Thiel	Catphan
120 kVp	0.84 / 0.84	B70	Thiel cadavers (3)	VGAS	Noise
0.9 pitch	2.11 / 2.05	I70/1	Catphan phantom		IQF_inv_
3 mm reconstruction thickness	4.19 / 4.08	I70/3			CNR
	6.37 / 6.18	I70/5			
	8.82 / 8.35				
	12.23 / 11.59				

### Image quality analysis

After acquisition, all data were sent to a PACS Workstation (GE Centricity PACS version 2.0 CRS5 SP2) for image quality assessment. Images were displayed on a 30-inch, 3-megapixel high-contrast color monitor (Barco MDCC 6130DL, Kortrijk, Belgium). The monitor was calibrated to comply with the DICOM Part 3.14 Greyscale Standard Display Function, using calibration software provided by the manufacturer (MediCal Pro, BARCO, Kortrijk, Belgium) [14]. Maximum luminance of all monitors was adjusted to 400 cd/m^2^ and ambient lighting levels were below 50 lux as recommended by AAPM TG 18 [15].

#### Scoring of Thiel images

Four experienced radiologists (PS: 25 years of experience; TDW, MVB and MV: 6 years of experience) assessed the chest CT scans and scored the image quality using criteria based on the European Guidelines on Quality Criteria for Computed Tomography [16]. The criteria are listed in Table 2. All criteria were evaluated in a predefined image area and a predefined image slice. For all three Thiel bodies, each stack was viewed individually and each structure was rated on a scale from 1 to 4 according to Table 3. An absolute VGA score (VGAS) for each reader was calculated as:

**Table 2:** Image quality criteria for chest CT

Criterion no.	Description:
1	Visually sharp reproduction of a nodular hypodense structure in a high density area such as an alveolus in consolidated lung parenchyma
2	Visually sharp reproduction of a nodular hypodense structure in a low density area such as normal lung parenchyma
3	Visually sharp reproduction of a nodular hyperdense structure in a low density area such as a vessel in aerated lung parenchyma
4	Visually sharp reproduction of an inter- or intralobular septum
5	Visually sharp reproduction of the bronchial wall
6	Visually sharp reproduction of the lung fissure
7	Visually sharp reproduction of a peripheral pulmonary artery branch
8	Visually sharp reproduction of fibrous strands
9	Visually sharp reproduction of the parietal and or visceral pleura

**Table 3:** Rating used to evaluate the clinical images

Rating	The structure in the image is:
1	Not visible
2	Poorly reproduced
3	Adequately reproduced
4	Very well reproduced

$$ \mathit{\mathsf{VGAS}}=\frac{{\displaystyle \sum_{\mathit{\mathsf{s}}=\mathsf{1}}^{\mathit{\mathsf{S}}}{\displaystyle \sum_{\mathit{\mathsf{t}}=\mathsf{1}}^{\mathit{\mathsf{T}}}{\mathit{\mathsf{G}}}_{\mathit{\mathsf{abs}},\mathit{\mathsf{s}},\mathit{\mathsf{t}}}}}}{\mathit{\mathsf{S}}*\mathit{\mathsf{T}}} $$ [17]

were G_abs,s,t_ is the rating for a particular structure (s) and Thiel body (t). S and T are the number of structures and Thiel body’s, respectively 9 and 3. The latter scoring reflected the image quality of the individual images without using a reference image [18].

All series were evaluated by the radiologists using Viewdex [19], a Java-based DICOM-compatible software tool for presentation and evaluation of images, without influencing the image quality. All images were blinded for acquisition and reconstruction parameters. The readers were allowed to adjust the image brightness and contrast and to magnify the images to full resolution. Viewdex defines a random order for each individual reader and all stacks were interpreted independently.

Before starting the study, a training session was organised to familiarise the readers with the scoring methodology.

#### Scoring of the Catphan phantom

Six medical physicists identified the minimally visible target diameter at three different contrast levels. The inverse image quality figure (IQFinv) was introduced for quantitative comparison of the contrast-detail images [20]. The inverse image quality figure is defined as

$$ \mathit{\mathsf{IQFi}}n\mathit{\mathsf{v}}=\frac{\mathsf{100}}{{\displaystyle {\sum}_{\mathit{\mathsf{i}}=\mathsf{1}}^n{\mathit{\mathsf{C}}}_{\mathit{\mathsf{i}}}{\mathit{\mathsf{D}}}_{\mathit{\mathsf{i}},\mathit{\mathsf{t}}\mathit{\mathsf{h}}}}} $$

where *D*_*i,th*_ denotes the threshold diameter for contrast *i* in mm and *C*_*i*_ denotes the contrast value. The higher the IQFinv, the better the low-contrast visibility. The IQFinv was calculated for all analysed images and averaged over the six readers.

The contrast to noise ratio relative to acrylic (soft tissue equivalent material) for teflon (bone equivalent material) was defined as:

$$ CNR=\frac{\left(RO{I}_t\mathit{\hbox{-}}\ RO{I}_a\right)}{S{D}_a} $$

where *ROI*_*t*_ is the mean attenuation for teflon, *ROI*_*a*_ the mean attenuation for acrylic and *SD*_*a*_ the mean noise for acrylic. CT attenuation values and mean noise (in Hounsfield units) for teflon and acrylic were obtained by manually placing a circular region of interest (ROI) of 200 pixels in the target materials. CNR’s were calculated in four consecutive slices of the Catphan CT number linearity and CT number accuracy module.

The image noise was evaluated using a circular ROI of 230 × 230 pixels in 11 following slices in the Catphan uniformity module.

### Statistical analysis

To determine the influence of different exposure and reconstruction settings, data were analysed using the Friedman test, a signed rank, non-parametric test used when comparing more than two related samples.

Inter-observer agreement for VGAS and IQF_inv_ values was determined by calculating the intraclass correlation coefficient. An intraclass correlation coefficient greater than 0.9 was considered to suggest an excellent inter-observer agreement [21].

After analysis of different fitting curves, a power function was selected as the best possible fit. Power functions are plotted for VGAS, noise, IQF_inv_ and CNR as a function of the mAs value. These curves are used to calculate the potential dose reduction when changing from a filtered back projection kernel (B70) at the clinically applied 90 refmAs to an iterative reconstruction kernel while maintaining the same value for noise, contrast-detail or CNR. To obtain a significant dose reduction, the two curves that are used, should differ significantly. This was examined by means of a Wilcoxon test, a signed rank, non-parametric test used when comparing two related samples. For this, all different readings (4, 11, 6, and 4 for VGAS, noise, IQFinv and CNR) for the six different mAs settings are considered which result in 24, 66, 36 and 24 data points for VGAS, noise, IQF_inv_ and CNR respectively.

A 95 % confidence interval was used for all statistical measures. All calculations were performed using the SPSS software tool (IBM SPSS statistics 22, IBM corp., NY, USA).

## Results

Excellent inter-observer agreement among the participating radiologists and among medical physicists was found by means of an intraclass correlation coefficient of 0.919 (*p* < 0.001) and 0.951 (*p* < 0.001) for the VGAS and IQF_inv_ parameters respectively. As a result, in the further analysis, scores averaged over the readers were used.

**Fig. 3:**
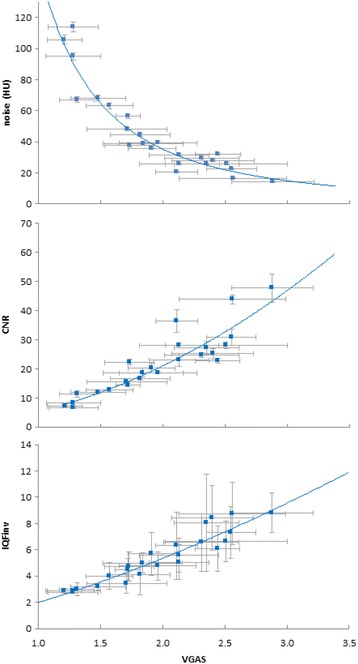
Mean noise, CNR and IQF_inv_ versus mean VGAS. The error bars in the x direction represent the standard deviation between the scores of the different radiologists. For noise, the error bars in the y direction represent the standard deviation between noise measurements in 11 following slices in the Catphan uniformity module. For CNR, the error bars in the y direction represent the standard deviation between CNR measurements in four consecutive slices of the Catphan CT number linearity and CT number accuracy module. For IQF_inv_, the error bars in the y direction represent the standard deviation between the six readers of the contrast-detail module in the Catphan phantom. Regression lines were plotted resulting in an r^2^ of 0.90, 0.88 and 0.92, *p* < 0.001, for noise, CNR and IQF_inv_ respectively

To evaluate the correlation between clinical and physical-technical image quality, regression curves were plotted for noise, CNR and IQF_inv_ as a function of VGA scores for the different refmAs settings (Fig. 3). Good correlation was found between noise and VGAS, 0.90, *p* < 0.001. A correlation coefficient of 0.88, *p* < 0.001 was obtained for CNR and VGAS. Contrast-detail (IQF_inv_) and VGAS resulted in a correlation coefficient of 0.92, *p* < 0.001.

To examine the influence of the iterative reconstruction strengths, the reconstruction settings mentioned in the materials and methods were applied to the Thiel images at 90 ref mAs. Catphan images acquired at mAs settings corresponding to 90 ref mAs were selected. A significant effect of the IR strengths was found for both the physical-technical and clinical image quality parameters (*p* < 0.05) except for IQF_inv_ (*p* = 0.706).

For both clinical and physical-technical image quality parameters as a function of the mAs value, a power function fit was applied for all types of kernels (noise and CNR r^2^ > 0.9, VGAS and IQF_inv_ r^2^ > 0.8, *p* < 0.05). As expected, for all 4 different types of reconstruction kernel , a significant effect of mAs settings was confirmed by means of a Friedman test for noise and contrast detail (*p* < 0.001) and for CNR (*p* < 0.05). Correspondingly, this influence was also found for VGAS (*p* < 0.05).

A significant difference was found between the curve of the B70 kernel and the curve of each strength of iterative reconstruction for all clinical and physical-technical image quality parameters, except for VGAS B70-I70/1.

**Fig. 4:**
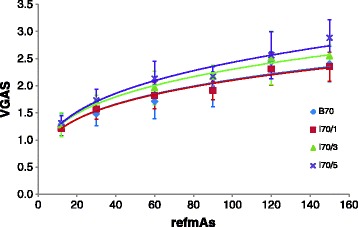
Mean VGAS versus refmAs for B70, I70/1, I30/3 and I70/5. The error bars in the y direction represent the standard deviation between the scores of the different readers. Power functions were plotted and for all kernels an r^2^ > 0.8 was obtained, *p* < 0.05

**Fig. 5:**
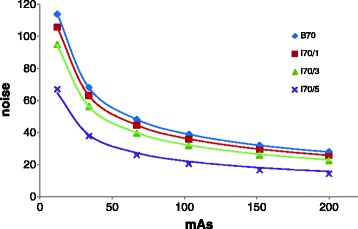
Mean noise versus mAs for B70, I70/1, I70/3 and I70/5. The error bars in the y direction represent the standard deviation between noise measurements in 11 following slices in the Catphan uniformity module. $\frac{a}{\sqrt{mAs}}$ regression curves were added

**Fig. 6:**
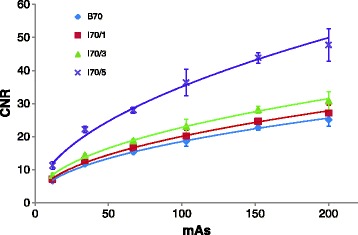
Mean CNR versus mAs for B70, I70/1, I70/3 and I70/5. The error bars in the y direction represent the standard deviation between CNR measurements in four consecutive slices of the Catphan CT number linearity and CT number accuracy module. Power functions were plotted and for all kernels an r^2^ > 0.9 was obtained, *p* < 0.05

**Fig. 7:**
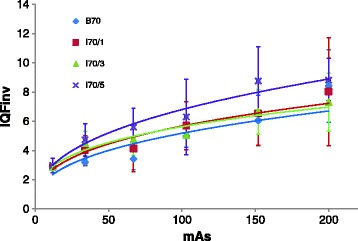
Mean IQF_inv_ versus mAs for B70, I70/1, I70/3 and I70/5. The error bars in the y direction represent the standard deviation between the scores of the different readers. Power functions were plotted and for all kernels an r^2^ > 0.8 was obtained, *p* < 0.05

The power function for VGAS, noise, CNR and IQF_inv_ as a function of refmAs settings is shown in Figs. 4, 5, 6 and 7. These curves were used to calculate the potential dose reduction when changing from a filtered back projection kernel at the clinically applied 90 refmAs to an iterative reconstruction kernel while maintaining the same value for noise, CNR or contrast-detail. In general, higher strengths of SAFIRE result in higher potential dose reduction. The potential dose reduction based on noise and CNR and IQF_inv_ varied from 14.0 to 37.8 %,16.0 to 71.5 % and 22.7 to 50.6 % respectively, depending on the strength of iterative reconstruction. Potential dose reduction based on clinical image quality varied from 27 to 37.4 % depending on the strength of iterative reconstruction. Consequently, the potential dose reduction is strongly dependent on the selected clinical or physical-technical parameter. From the physical-technical image quality parameters, dose reductions based on IQF_inv_ correspond best with dose reductions based on VGAS The results are summarized in Table 4.

**Table 4:** Potential dose reduction for different clinical and physical image quality parameters and different iterations steps

	Potential dose reduction (*p*-value)			
Reconstruction kernel	VGAS	Noise	IQF_inv_	CNR_teflon_
I70/1	0 % (0.887)	14.0 % (<0.001)	22.7 % (0.034)	16.0 % (<0.001)
I70/3	27.0 % (0.021)	31.4 % (<0.001)	23.0 % (0.031)	35.8% (<0.001)
I70/5	37.4 % (0.001)	67.8 % (<0.001)	50.6 % (<0.001)	71.5 % (<0.001)

## Discussion

Methods for patient dose evaluation are easily available but techniques for objective clinical image quality optimisation are far more complicated. VGA and ROC studies are commonly used to assess clinical image quality [22]. In VGA studies, a relative or absolute scoring is performed based on the visibility of normal anatomical structures [8]. The task for observers in a ROC study is to detect whether a patient’s image contains a pathological structure or not [9]. However, these studies are difficult to implement in routine practice since they imply a significant additional workload for the radiologists and large patient data groups must be available. Therefore, the latter methods are not feasible within a routine quality assurance programme.

A more practical approach to assess the image quality is the use of physical-technical phantoms, such as the Catphan phantom, where physical-technical parameters such as noise, CNR, MTF and contrast-detail can be analysed. Such physical-technical phantoms have been widely used for the objective analysis of the image quality performance of CT systems [23]. Catphan studies are easily implemented in a quality assurance programme since no patient data are required and images can be analysed by the medical physics expert. However, the disadvantage of the Catphan phantom is the uniform background. Actual patient images are clearly not uniform and contain detailed anatomical features and textures. These background anatomical textures can influence image quality, both because the presence of anatomical texture affects observer performance and quantum noise [7].

In present study, a VGA and Catphan study was set up to assess the relationship between physical-technical and clinical image quality in chest CT examinations, using Thiel embalmed cadavers and the Catphan@504 phantom. In contrast to conventional embalming procedures using formalin for conservation, this new technique results in a very well preservation of the lung structures [11, 12]. To approximate as good as possible the normal patient anatomy, Thiel bodies were ventilated during image acquisition [13]. After assessment of different thoracic regions by experienced radiologists, it was confirmed that Thiel bodies can be applied to assess clinical image quality using VGA and ROC studies.

Recently, there is growing interest in developing and utilising model observers to accurately predict human observer performance for image system optimization and comparison. A model observer is a mathematical model that can be used to predict human detection performance for some specific imaging tasks [24]. A variety of models, which differ in how much information about signal and noise are used and whether certain properties of the human visual system responses are incorporated, have been proposed and applied to medical image research [24, 25]. However, up till now, phantom images and simulated lesions are used to assess these models. How much real lesions and anatomical backgrounds affect model observer performance remains under investigation [26]. Possibly, the concept of Thiel embalmed cadavers could be used to help validate model observer applications.

Although good correlation was found between physical-technical image quality parameters (noise, CNR and contrast-detail) and clinically observed quality as scored by radiologists (VGAS), the potential dose reduction based on the physical-technical image quality parameters noise and CNR, is much higher compared to the potential dose reduction based on the clinical image quality. This overestimate of the dose reduction can be explained because the uniform phantom does not account for the complex relationship between anatomical variability and image quality. On the contrary, the potential dose reduction based on IQF_inv_ is more consistent with the potential dose reduction based on VGAS. However the measurements are very crude using the Catphan phantom as only three contrast levels are present. Optimisation of a contrast-detail phantom for CT is necessary and could give added value to the concept of contrast-detail analysis in CT image quality studies similar to the use of contrast-detail phantoms in mammography and conventional radiology [27, 28].

While this study illustrates that noise measurements in uniform backgrounds are not ideal to assess the effect of iterative reconstruction, a large part of the literature is still based on this technique. Mieville et al. [29] used the Catphan phantom to assess noise, CT number accuracy, noise power spectrum and MTF at varying CTDI values for both FBP images and IR images. Milim Kim et al. [30] used the phantom of the American College of Radiology, a solid water phantom with 5 imbedded test objects to evaluate image noise, SNR and CNR. No comparisons were made between the possible dose reduction based on the different parameters. Ghetti et al. [31, 32] assessed image noise in a uniform water phantom. Since noise reduction in these studies are based on uniform phantoms, it is questionable if these results are applicable in clinical practice. The nonlinear nature of IR methods has also introduced significant challenges to the characterization of spatial resolution performance. In this framework, Li et al. introduced a concept of task specific measurements of the spatial resolution by locally measuring the point spread function for a given feature of interest at a given radiation dose level in an anthropomorphic phantom [33].

Other studies performed clinical image quality assessment on patient data, which automatically limits the amount of dose settings that can be used. Exact calculation of the potential dose reduction without loss of image quality is thereby impossible. Prakash et al. [34] scanned 54 patients at a mean effective dose of 12.2 mSv reconstructed with FBP and 98 patients at a mean effective dose of 8.9 mSv reconstructed with an iterative reconstruction technique (30 % ASIR, GE) resulting in a mean dose reduction of 27.6 %. All chest CT examinations were scored diagnostically acceptable. Pontana et al. [35] scanned 80 patients two times with constant CT parameters except for the refmAs which was decreased by 30 %. High dose chest CT images were reconstructed with FBP, low dose chest CT images were reconstructed with an iterative reconstruction technique (IRIS algorithm, Siemens). There was no significant difference in objective noise, CNR, SNR and overall subjective image quality between the two groups. In both studies, physical-technical as well as clinical image quality was assessed. However, no further correlation analysis was made between the physical-technical and clinical image quality. Since only two dose settings were examined, the dose reduction based on clinical and physical-technical image quality was identical. Consequently, the maximum potential dose reduction without loss of image quality, could not be assessed and no conclusions can be made about the discrepancy in potential dose reduction when using physical-technical parameters rather than clinical image quality assessment.

There are several limitations of our study. Firstly, the available Thiel cadavers all had a BMI between 20 and 25. It is possible that the correlation between clinical and physical-technical image quality and the effect of iterative reconstruction can be influenced by patient size. Secondly, clinical image quality was assessed on unenhanced CT images. Possibly the correlation and potential dose reduction can be affected when contrast agents are used. Thirdly, clinical image quality was assessed by a subjective overall quality score and not by means of detection of pathology. Detection of lesions by means of a receiver operating characteristic (ROC) analysis could give a more precise assessment of image quality for a specific clinical application.

## Conclusions

In summary, our results demonstrate that noise assessments in a uniform phantom overestimate the potential dose reduction for the SAFIRE IR algorithm. Since the IQF_inv_ based dose reduction is quite consistent with the clinical based dose reduction, an optimised contrast-detail phantom could improve the use of contrast-detail analysis for image quality assessment in chest CT imaging. In conclusion, one should be cautious to evaluate the performance of CT equipment taking into account only physical-technical parameters as noise and CNR, as this might give an incomplete representation of the actual clinical image quality performance.
